# Epidural Cystic Spinal Meningioma: A Case Report

**DOI:** 10.1097/MD.0000000000003030

**Published:** 2016-03-18

**Authors:** Ji Zhang, Zheng-he Chen, Zi-feng Wang, Peng Sun, Jie-tian Jin, Xiang-heng Zhang, Yi-ying Zhao, Jian Wang, Yong-gao Mou, Zhong-ping Chen

**Affiliations:** From the Department of Neurosurgery (JZ, Z-HC, X-HZ, Y-YZ, JW, Y-GM, Z-PC); Department of Anesthesiology (PS); Department of Pathology (J-JT); Department of Experimental Research, State Key Laboratory of Oncology in South China, Sun Yat-sen University Cancer Center, Guangzhou, China (Z-FW).

## Abstract

Cystic spinal meningioma (CSM) is an uncommon meningioma variant. Extradural CSMs are particularly rare and difficult to distinguish from other intraaxial tumors. This study presents a case of a 36-year-old woman with intraspinal extradual CSM at the thoracolumbar spine. She experienced persistent weakness, progressive numbness, and sensory disturbance in the right lower limb. Magnetic resonance imaging (MRI) of the patient revealed an irregular cystic mass at the thoracic 11 to lumbar 3 levels dorsally. This case was misdiagnosed as other neoplasms prior to surgery because of the atypical radiographic features and location of the tumor.

Extradural CSMs should be considered in the differential diagnosis of intraspinal extradural cystic neoplasms. Complete removal of cystic wall provides an optimal outcome, rendering the lesion curable.

## INTRODUCTION

Among meningiomas of the central nervous system, cystic meningiomas account for an extremely low proportion and seem to have a predilection for children.^1^ Cystic spinal meningiomas (CSMs) are easily confused with other tumors with cyst formation and have rarely been reported in the literature. The 1st known case of special CSM was entirely extradural. Characteristic radiological investigations are the basis of preoperative diagnosis. However, when these lesions are almost cystic in nature or have cystic components, the radiological appearance can be confusing due to its rarity. In this report, we describe an unusual case of thoracolumbar epidural cystic meningioma in a young patient to provide additional data for this meningioma variant and to report intraoperative findings and our experience of management.

## CASE REPORT

A 36-year-old woman was referred to our institution and presented with a 2-year history of persistent weakness and progressive numbness in the right lower limb accompanied by newly developed sensory disturbance. On neurological examination, she was found to have low strength in her right lower extremity (4/5), right hamstring strength of 4/5, and right quadriceps and iliopsoas strength of 3/5. Her left foot dorsiflexion, her right plantar flexion, and knee flexion were normal. The remainder of the left lower limb showed normal strength. There was decreased sensation in the right L-1 and 5 distribution and the left L-1 distribution. The results of laboratory evaluation were unremarkable. The patient was in good health and had no family history of any inheritable neoplastic disease.

**FIGURE 1:**
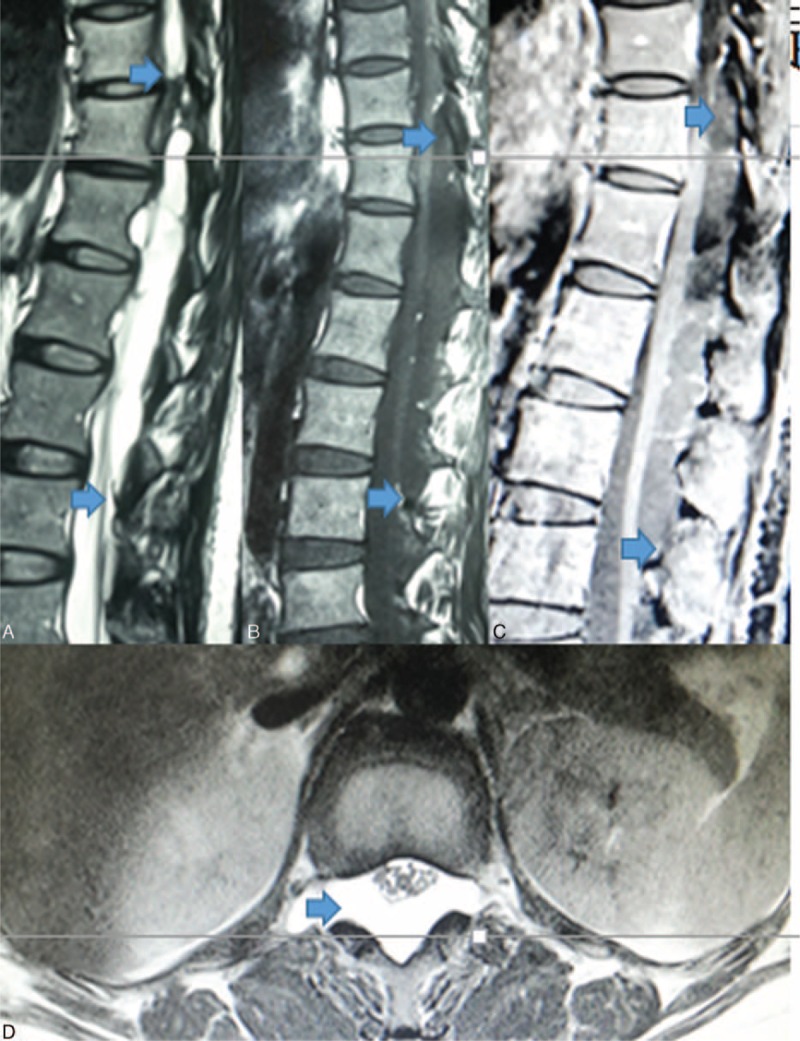
Preoperative sagittal T2-weighted magnetic resonance imaging (MRI) (A), T1-weighted MRI (B), T1-weighted MRI with gadolinium enhancement (C), and axial T2-weighted MRI (D) revealed a mixed signal mass with snake-like shape and fishnet-enhancement at the level of T11 to L3 level. Both T1- and T2-weighted MR images show a low and high signal like cerebrospinal fluid.

A multiparametric magnetic resonance imaging (MRI) of the head and whole spinal cord was conducted using a high-field (1.5T) resonator. MRI of the head illustrated no mass lesion. Conventional sequences without and with gadolinium enhancement revealed a heterogeneously irregular cystic mass from thoracic 11 to lumbar 3 dorsally. The lesion was a well-circumscribed extradural lesion with a snake-like shape. This mass was hypointense on T1-weighted images, hyperintense on T2-weighted images, and contrast enhancement in the cystic wall with the mural nodule (Figure 1A–D). Tiny tumoral parenchyma that paralleled the level of thoracic 11 was identified in the upper pole of the mass, which had multiple small cysts with same signals. The dural tail sign was not noticed, and the decompression of the spinal marrow was noted. These MRI findings were highly suggestive of an enterogenous cyst or arachnoid cyst.

The patient in the prone position underwent a total laminectomy of T11 to L3 via a conventional posterior midline approach. After the vertebral plate was opened, the epidural mass was identified with clear cystic appearance, connected to the nerve roots, and tightly adhered to the dorsal dura in the surgical field. The adjacent dura and nerve root sleeves were slightly thickened. The cystic wall was milky white, thin, and similar to the arachnoid; and the cystic liquid was pale yellow and collected for test. Several separated cystic cavities were observed after opening the tumor. The extradural cystic wall was separable from the adjacent dura. The cystic walls were completely ablated with coagulation of the dural attachment using a bipolar coagulator. Under the operative microscope, the mass was totally removed. A bone plasty was performed to maintain structural stability, followed by skin closure.

**FIGURE 2:**
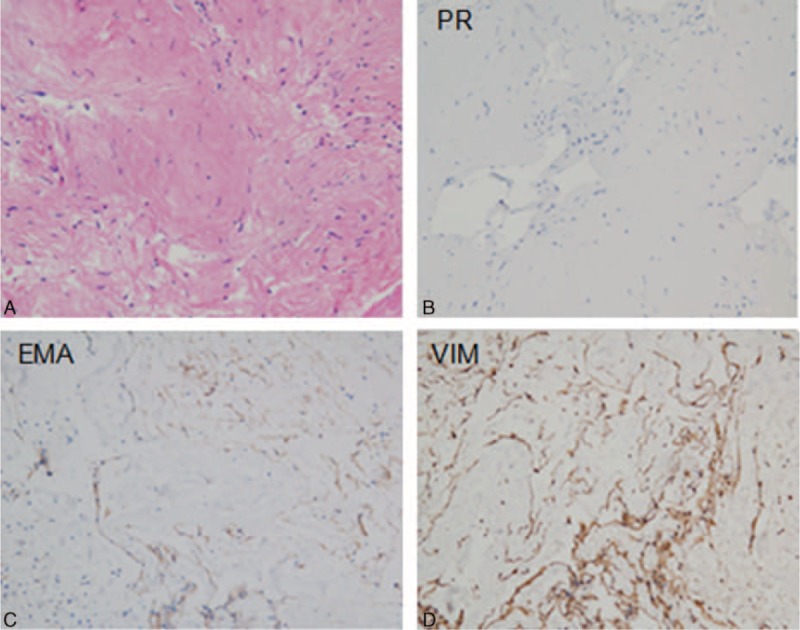
Histopathologic section of the resected tumor demonstrated whorls of meningiothelial cells with indistinct cell borders (hematoxylin and eosin staining, ×200) (A). Immunohistochemistry shows EMA (B), PR (C), and VIM (D) are positive (×100). These findings were compatible with cystic meningioma. EMA = epithelial membrane antigen, PR = progesterone receptor, VIM = vimentin.

**FIGURE 3:**
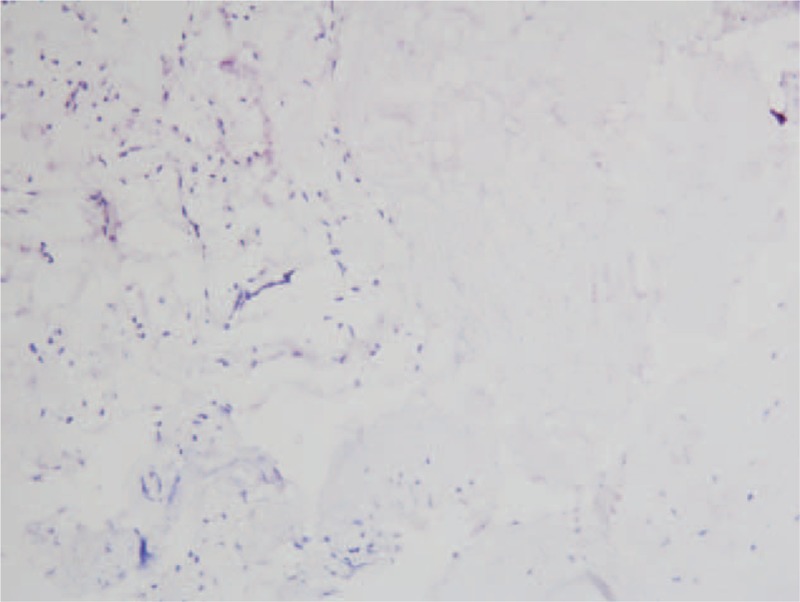
Immunohistochemistry shows P53 is negative (×100).

Pathological examination demonstrated that these cystic walls had a glabrous internal surface. Microscopically, the wall was composed of largely uniform spindle-shaped cells in a collagen-rich matrix. Whorl formation was conspicuous with scanty eosinophilic cytoplasm, with nuclear hyperchromasia (Figure 2A). The tumor had a low level of proliferative activity and the Ki-67 labeling index of the tumor was less than 1%. On immunohistochemical analysis, the cells were positive for epithelial membrane antigen, progesterone receptor, and vimentin (Figure 2B–D). It does not express glial fibrillary acid protein (GFAP), S100, cytokeratin (CK), Des, HHF35 (data not shown), and P53 (Figure 3). Histopathological studies confirmed the mass to be a meningioma (World Health Organization, WHO grade I).

**FIGURE 4:**
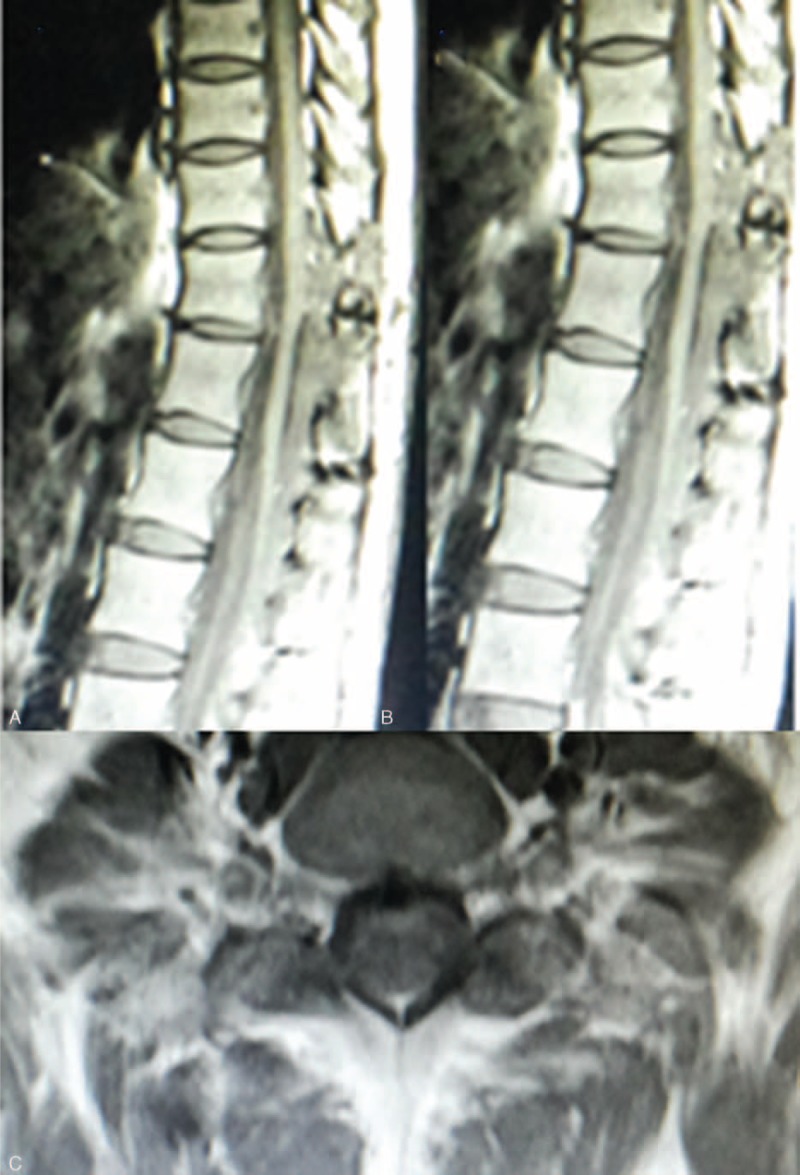
Postoperative sagittal T1-weighted (A), T1-weighted enhanced with gadolinium (B), and axial T2-weighted (C) magnetic resonance (MR) images demonstrated no mass residual.

The postoperative outcome was favorable with no neurological deficit. She progressively showed neurological recovery during the postoperative course and resumed her normal life without additional treatment because of the tumor's benign nature and satisfactory excision. Residual tumor tissue was not identified on regular follow-up MRI (Figure 4A–C).

## DISCUSSION

Spinal meningiomas are common extra-axial solid lesions with distinguishing features, and most of them have a thoracic localization.^2^ Cystic meningiomas in the central nervous system, predominantly occurring in the 4th ventricle, cerebral convexity, and parasagittal areas, possess some deceptive traits which create a diagnostic dilemma.^3,4^ There are limited reports regarding extradural cystic spinal meningiomas in the English literature. Interestingly, our case was almost absolutely cystic with an epidural location in the thoracolumbar spine.

Spinal meningiomas tend to be isointense on T1- and T2-weighted images, enhancing strongly after administration of Gd-DTPA. CSMs with atypical features complicate the imaging workup and cannot be easily identified on computer tomography scan and MRI due to their rarity. The thickening of the adjacent dura on MRI, which is highly useful for the diagnosis of cystic meningioma, was not observed in the case. The mass was misdiagnosed as other epidural tumors due to its location, which would more likely represent an enterogenous cyst or arachnoid cyst, whose intensity on MRI did not enhance due to its unusual imaging appearance with predominantly cystic features while presenting the histologic finding of calcifications supporting the diagnosis of meningioma. The enhancement of the tiny mural nodule at the upper end and of the peripheral cystic wall infiltrated by tumor cells corresponded to the characteristic of cystic meningioma. This is a potentially important diagnostic sign that raises the suspicion of cystic meningiomas.

Preoperative differentiation between extradural CSM and other intraspinal fluid-filled neoplasms mentioned below is extremely difficult and frequently carried out after pathological examination.^5^ Ours is the 1st reported case of a virtually intraspinal CSM in the epidural space. Cystic neoplasms have been used clinically to differentiate neurilemmomas from enterogenous cysts within the spine. Precise diagnosis is extremely crucial for making preoperative plan because the result affects the surgical strategy. A snake-like cystic mass in the dorsal epidural region corresponds with schwannoma. Its cystic component had no enhancement, and the enhancement presented a spider-web formation and a mural nodule on gadolinium-contrast MR image. Schwannoglioma or enterogenous cysts, which are the most common tumor types that appear with cysts, were interpreted preoperatively, due to their imagenologic features. Typical radiological characteristics of cystic spinal meningiomas are exceedingly similar to schwannomas, metastatic tumors, enterogenous cysts, and hydatid cysts.^6^ However, conclusive result after surgery overthrew our prospective judgment. The pathological results also assured us that the cyst wall consisted of tumor cells.

As far as the pathological types of cystic meningomas are concerned, they are preferable to be atypical, and their biologic behaviors are more aggressive and demonstrate an increased risk of recurrence after initial treatment.^7^ However, our case was benign presenting atypical biologic behavior. Subsequent radiation for patients with cystic mengingomas depends to a great extent on the histopathologic features. A low incidence of recurrence exists following complete resection. In our patient, no follow-up treatment was considered due to the favorable further and benign nature of the lesion. The follow-up interval should be short for aggressive cystic meningiomas, which is useful for the early detection of the residual and recurrence, and for guiding subsequent treatment.

MRI with and without contrast was performed to identify the margin of the tumor and the range and then work out the surgical resection plans. During the surgical procedure of tumor removal, it is arduous to control contiguous normal tissue bleeding. After complete excision, the errhysis was apparent due to the number of tiny veins around the tumor. Fluid gelatin was quite useful for restraining hemorrhage. The cyst wall should be removed completely, because the cyst walls display contrast enhancement and are positive for tumor cells assessed by intraoperative biopsy. If not, it can give rise to the risk of recurrence. The literature that reported cystic meningiomas spread through the cystic fluid to the wall of the cyst, but there was no affirmative conclusion that tumor recurrence was significantly correlated with cystic fluid spread.^8^ In our case, the result of biopsy for cyst liquid was obtained and the tumor cells were not observed under microscopic examination.

Cystic meningiomas are dissolved into intratumoral and peritumoral cysts.^8^ Preoperative diagnosis is still difficult for neurosurgeons to date. To the best of our knowledge, there is no pure CSM described without distinct parenchyma in the published literature. Owing to the vast majority of cystic involvement, classic image features were absent, which radiates in flurried manner as our case. The typically differential diagnosis should involve neurilemmoma with cystic formation, metastases, and enterogenous cyst.^6^ The literature represents some theories on the mechanism of cyst formation in this entity. The underlying etiology of cyst formation remains obscure.^9^ Some theories such as central necrosis, active secretion of fluid by tumor cells, and evolution of cerebral edema were involved in many hypotheses.^10^

## CONCLUSION

We describe an uncommon case of CSM localized in an extradural site. Making a correct preoperative diagnosis remains challenging because of their rarity and affinity to other types of tumors with cystic changes. This type of clinical entity should be considered in the differential diagnosis of intraspinal extradural neoplasms. Complete removal of cystic wall provides an optimal outcome, rendering the lesion curable.
